# Interactions between occupational stress, compassion fatigue, and work engagement among nurses

**DOI:** 10.1590/0034-7167-2024-0555

**Published:** 2025-11-07

**Authors:** Luciano Garcia Lourenção, Francielle Garcia da Silva, José Gustavo Monteiro Penha, Daniela Menezes Galvão, Vagner Ferreira do Nascimento, Natália Sperli Geraldes Marin dos Santos Sasaki, Francisco Rosemiro Guimarães Ximenes, Luiz Antônio Alves de Menezes

**Affiliations:** I Universidade Federal do Rio Grande. Rio Grande, Rio Grande do Sul, Brazil; II Universidade do Estado do Mato Grosso. Barra do Bugres, Mato Grosso, Brazil; III Faculdade de Medicina de São José do Rio Preto. São José do Rio Preto, São Paulo, Brazil; IV Universidade Estadual Vale do Acaraú. Sobral, Ceará, Brazil; V Universidade Federal de Ouro Preto. Ouro Preto, Minas Gerais, Brazil

**Keywords:** Occupational Stress, Compassion Fatigue, Work Engagement, Nurses, Hospitals., Estrés Ocupacional, Desgaste por Empatía, Compromiso en el Trabajo, Enfermeros y Enfermeras, Hospitales.

## Abstract

**Objectives:**

to analyze the levels of compassion fatigue, occupational stress, and work engagement, and their correlation among nurses at a university hospital.

**Methods:**

cross-sectional study conducted in 2019 with 83 nurses, using validated scales. Descriptive, reliability, and correlation tests were applied (p ≤ 0.05).

**Results:**

a prevalence of 34.9% for occupational stress and 15.9% for compassion fatigue was observed; high levels of dedication (4.6 ± 1.0), absorption (4.2 ± 1.0), and vigor (4.4 ± 1.1) were reported. Nurses with occupational stress were more likely to experience compassion fatigue (OR = 2.668; 95% CI: 0.799-8.898), and had higher levels of burnout (mean = 53.8; p = 0.013) and secondary traumatic stress (mean = 53.0; p = 0.047). Professionals with compassion fatigue showed reduced dedication (p = 0.011) and vigor (p = 0.001).

**Conclusions:**

the relationship between occupational stress and compassion fatigue increases the risk of burnout and secondary traumatic stress, and reduces dedication and vigor, highlighting the need for institutional measures to promote well-being, especially in the post-pandemic context marked by overload, resource scarcity, and distress.

## RESUMEN

**Objetivos:**

analizar los niveles de fatiga por compasión, estrés ocupacional y compromiso laboral, así como su correlación, en enfermeros de un hospital universitario.

**Métodos:**

estudio transversal realizado en 2019 con 83 enfermeros, utilizando escalas validadas. Se aplicaron pruebas descriptivas, de confiabilidad y de correlación (p≤0,05).

**Resultados:**

se observó una prevalencia del 34,9% de estrés ocupacional y del 15,9% de fatiga por compasión; altos niveles de dedicación (4,6±1,0), absorción (4,2±1,0) y vigor (4,4±1,1). Los enfermeros con estrés ocupacional presentaron mayor probabilidad de desarrollar fatiga por compasión (OR = 2,668; IC95%: 0,799-8,898), niveles elevados de burnout (media = 53,8; p = 0,013) y estrés traumático secundario (media = 53,0; p = 0,047). Los profesionales con fatiga mostraron una reducción en los niveles de dedicación (p = 0,011) y vigor (p = 0,001).

**Conclusiones:**

la relación entre el estrés ocupacional y la fatiga por compasión incrementa el riesgo de burnout y de estrés traumático secundario, y reduce la dedicación y el vigor, lo que resalta la necesidad de medidas institucionales para promover el bienestar, especialmente en el contexto pospandémico, caracterizado por la sobrecarga laboral, la escasez de recursos y el sufrimiento.

## INTRODUCTION

The hospital environment presents physical and psychosocial challenges for nurses, who deal with patient suffering, heavy workloads, and pressure to make rapid and accurate decisions. These situations can trigger compassion fatigue and stress, compromising engagement and affecting both the well-being of professionals and the quality of services^(1,2)^.

Compassion fatigue results from physical, emotional, and mental exhaustion associated with caring for individuals in intense suffering, causing biological, psychological, and social dysregulation^(3)^. According to the Professional Quality of Life model, it occurs when high levels of burnout and secondary traumatic stress are combined with low compassion satisfaction (CS). While burnout reflects exhaustion and inefficiency, secondary traumatic stress stems from continuous exposure to suffering, leading to sleep disturbances and distressing memories. Conversely, CS reflects the pleasure derived from helping others in a meaningful way^(2,4)^.

In Brazil, compassion fatigue has been observed among primary care nurses^(5)^ and professionals in critical care units, with a higher incidence among nursing technicians, women, and younger professionals^(4)^.

Occupational stress refers to the physical, emotional, and behavioral responses to excessive work demands^(6)^. When prolonged, it can result in anxiety, depression, psychosomatic disorders, and can negatively affect quality of life and professional performance^(7,8)^. This biopsychosocial condition is complex and raises significant concern regarding the health of professionals, especially due to its high prevalence among nurses^(6,7)^.

Work engagement is essential for understanding professional motivation and performance, representing a positive mental state characterized by vigor, dedication, and absorption in work activities. This concept is directly linked to positive outcomes for both workers and organizations. Vigor reflects the energy and enthusiasm professionals feel while performing their duties, while dedication involves a strong sense of purpose and motivation. Absorption, in turn, refers to complete immersion in tasks, in which the professional loses track of time, demonstrating high concentration. These three dimensions interact with one another and are essential indicators of employee well-being, impacting service quality and organizational effectiveness^(6,9)^.

The development of compassion fatigue and occupational stress can impact nurses’ work engagement^(2,7)^, making the assessment of these conditions essential. Research is crucial to understanding the challenges faced by these professionals and to developing strategies that prevent and address these issues. Although these conditions are common in the global healthcare sector, factors such as location, hospital department, and low investment in healthcare significantly influence the increase or reduction of these problems. Furthermore, difficulties already present before the COVID-19 pandemic-such as precarious working conditions, excessive workloads, exhaustion, and suffering-were exacerbated during the pandemic. The fear of facing an unknown disease also directly affected nurses’ mental and physical health^(2,10)^.

In addition, issues such as low wages and lack of professional recognition, which have long affected these workers, may have been intensified during the pandemic^(11)^. Therefore, it is relevant to develop local studies in different hospital institutions, considering the specific characteristics of each context. These studies can support the development of more targeted, adequate, and effective coping measures to improve working conditions and nurses’ well-being.

Based on the above, the guiding question of this study was: What is the correlation between occupational stress, compassion fatigue, and work engagement among nurses working in hospital settings? The hypothesis adopted is that nursing professionals working in hospital units experience occupational stress and compassion fatigue, which may reduce their levels of work engagement.

## OBJECTIVES

To analyze the levels of compassion fatigue, occupational stress, and work engagement and their correlation among nurses in a university hospital.

## METHODS

### Ethical aspects

In compliance with ethical and legal principles related to research involving human subjects, the study was approved by the Research Ethics Committee of the Federal University of Rio Grande.

### Study design, period, and setting

This was a cross-sectional, descriptive, and correlational study conducted between September and December 2019 with nurses working at a medium-sized university hospital affiliated with the Brazilian Hospital Services Company (EBSERH) and located in the far south of Brazil. The Strengthening the Reporting of Observational Studies in Epidemiology (STROBE) tool was used to guide the research.

### Population and sample: inclusion and exclusion criteria

The study population consisted of all nurses who had been working at the university hospital for at least six months. This period was established by the researchers based on the understanding that six months would be sufficient for professionals to become familiar with the work environment. Nurses who were on medical leave or vacation during the data collection period were excluded, resulting in an estimated population of 141 nurses.

The sample was selected by convenience, a method justified by the accessibility and availability of participants, which allowed for practical and feasible data collection within the time frame defined for the study. This approach enabled the inclusion of a significant proportion of the institution’s nursing staff, ensuring representativeness of the university hospital nursing team. All professionals were invited to participate, and those who returned completed instruments were included in the final sample, which consisted of 83 nurses (58.9% of the population). There were no formal refusals to participate in the study.

### Study protocol

Four self-administered instruments were used: a questionnaire on sociodemographic and professional characteristics (such as academic background, sex, age, income, marital status, employment status, work shift, time in practice, hours of sleep, additional jobs, and physical activity); the Work Stress Scale (WSS)^(12)^; the Brazilian version of the Professional Quality of Life Scale (ProQOL-BR)^(13)^; and the short version of the Utrecht Work Engagement Scale (UWES-9)^(14)^.

The WSS, composed of 23 items, assesses work-related stress situations. Its validation in Brazil showed high internal consistency (α = 0.91; KMO - Kaiser-Meyer-Olkin = 0.91)^(6,12)^. The ProQOL-BR measures CS, Burnout (BO), and Secondary Traumatic Stress (STS), with 30 items distributed across three categories (10 items each). The Brazilian version demonstrated good factorability (KMO = 0.861) and internal consistency (CS = 0.81; BO = 0.83; STS = 0.76)^(4,13)^.

The UWES-9 assesses work engagement through the dimensions of Vigor, Dedication, and Absorption, and has shown robust psychometric properties in Brazil (α = 0.93; CFI- Comparative Fit Index = 0.97; TLI - Tucker-Lewis Index = 0.98)^(9,14)^.

Data were collected in person by a trained graduate-level nurse. Professionals were approached in their work units, either individually or in groups. The study objectives were presented, and participants were invited to take part in the study; they received the informed consent form (ICF) and the data collection instruments. A deadline was set for returning the completed instruments by the next work shift. After the deadline, the graduate student returned to the units to collect the completed, anonymous instruments. If professionals forgot or reported not having had time to respond, the deadline was extended to the following shift.

To minimize sample loss and avoid selection bias, strategies such as personal invitations during work shifts and flexible deadlines were adopted, allowing professionals to complete the questionnaires by the next shift, with additional extensions if necessary. These measures aimed to increase participation and reduce the impact of sample loss.

### Data Analysis and Statistics

Data were entered twice to detect inconsistencies or errors. To assess whether the data followed a normal distribution, the Kolmogorov-Smirnov test was used, with the following results: Occupational Stress (p = 0.470), Dedication (p = 0.230), Absorption (p = 0.326), Vigor (p = 0.400), CS (p = 0.061), BO (p = 0.788), and STS (p = 0.374). Reliability analysis of the construct measures indicated that Cronbach’s alpha values ranged from 0.70 to 0.92, demonstrating the reliability of the results^(9)^. Sociodemographic and professional variables were used to describe the nurses’ profiles.

To evaluate occupational stress, the scale score was calculated as the average of the 23 items. This score ranges from 1 to 5, and as recommended by the instrument, higher values indicate greater stress levels. Mean scores equal to or greater than 2.5 indicate a significant level of stress^(6)^. Subsequently, the variable was dichotomized as “with stress” (WSS ≥ 2.5) or “without stress” (WSS < 2.5).

Compassion fatigue was assessed through the general scores of the ProQOL-BR subscales^(2,4)^. Raw scores for the ProQOL-BR dimensions (CS; BO; and STS) were converted into z-scores using descriptive analysis and selecting the option “save standardized values as variables.” Next, the z-scores were converted into t-scores using the formulas: (ZSC*10)+50; (ZBO*10)+50; (ZSTS*10)+50, enabling comparison among the three dimensions^(4)^.

The subscale scores were then computed and categorized according to the 75th percentile of the score distribution. Professionals who scored at or above the 75th percentile on each subscale were considered to have a high level of BO or secondary traumatic stress. Compassion fatigue was subsequently defined as the combination of high BO and high STS levels^(4)^.

To assess work engagement, scores from the UWES dimensions were calculated following the statistical model proposed in the UWES Preliminary Manual. The mean and standard deviation were presented for each dimension of the scale. After calculating the scores, values were classified based on the manual’s decoding: scores from 0 to 0.99 indicated very low engagement; 1 to 1.99, low engagement; 2 to 3.99, moderate engagement; 4 to 4.99, high engagement; and 5 to 6, very high engagement^(9)^.

The association between occupational stress and compassion fatigue was assessed using odds ratios (OR) and Fisher’s exact test. Comparisons of mean scores for the different scale dimensions were performed using Student’s t-test. The level of significance adopted was 5% (p ≤ 0.05).

Pearson’s correlation test (r) was applied to examine the correlation among the values of different scales. Correlations were considered weak for r values up to 0.39, moderate for values between 0.40 and 0.69, and strong for values equal to or greater than 0.70^(9)^.

## RESULTS

Among the 83 professionals evaluated, the majority were female (75.9%), aged 26 to 35 years (50.6%), married or in a stable union (57.8%), public employees with statutory contracts (97.6%), and had a household income of two to five minimum wages (57.1%). Most professionals performed both administrative and care-related tasks (49.4%), worked predominantly the night shift (38.6%), engaged in physical activity (62.7%), reported sleeping six to eight hours per day (68.7%), and had between two and ten years (39.8%) or between ten and twenty years (32.1%) of professional experience (Table 1).

**Table 1:** Sociodemographic and professional characteristics of nurses, Rio Grande, Rio Grande do Sul, Brazil, 2023 (N = 83)

Variables	n	%
Sex		
Male	20	24.1
Female	63	75.9
Age group (years)^*^		
26-35 (professional training)	42	50.6
36-50 (professional maturity)	29	34.9
51-60 (professional slowdown)	6	7.2
61 or older (retirement)	1	1.2
No response	5	6.0
Marital status		
Married / In a stable union	48	57.8
Single	22	26.5
Divorced / Separated	8	9.6
No response	5	6.0
Type of employment relationship		
Statutory public servants	81	97.6
Employees under the Consolidation of Labor Laws (CLT)	2	2.4
Household income (minimum wages^**^)		
Two to five	7	8.4
Six to tem	56	67.5
More than tem	20	24.1
Type of activity		
Care-related	30	36.4
Administrative	12	14.5
Administrative and care-related	41	49.4
Work shift		
Morning	19	22.9
Afternoon	19	22.9
Night	32	38.6
Full-time day	13	15.7
Practices physical activity		
Yes	52	62.7
No	31	37.3
Daily hours of sleep		
Less than 6	23	27.7
6 to 8	57	68.7
More than 8	3	3.6
Length of professional experience		
≤ 2 years	6	7.2
> 2 and ≤ 10 years	33	39.8
> 10 and ≤ 20 years	26	32.1
> 20 years	14	16.9
No response	4	4.8

* Age group classification according to Machado et al., 2016(15);

** Minimum wage value: R$ 998.00 / USD 236.29 (1 USD = R$ 4.2235).

Cronbach’s alpha coefficient indicated that the WSS showed high reliability (α = 0.92). The results showed that 29 nurses (34.9%) had scores indicative of significant stress (≥ 2.5).

In the assessment of professional quality of life (Table 2), it was observed that 24.4% of nurses presented a high level of CS, 25.6% had a high level of BO, and 26.8% a high level of secondary traumatic stress. Conversely, 24.4% had a low level of CS, 50.0% a moderate level of BO, and 46.3% a moderate level of secondary traumatic stress. In addition, 13 nurses (15.9%) presented compassion fatigue (high level of BO combined with a high level of secondary traumatic stress).

**Table 2:** Cutoff points of the ProQOL-BR and frequencies of nurses by classification level in the dimensions of professional quality of life, Rio Grande, Rio Grande do Sul, Brazil (N = 82)^*^

ProQOL-BR Dimensions^**^	Cronbach’s Alpha	Cutoff Points - Percentiles (t-scores)			Classification Levels n (%)		
		25	50	75	Low	Medium	High
Compassion Satisfaction	0.84	42.9	50.5	58.0	20 (24.4)	42 (51.2)	20 (24.4)
Burnout	0.71	42.8	48.8	58.0	20 (24.4)	41 (50.0)	21 (25.6)
Secondary Traumatic Stress	0.77	42.8	50.0	58.6	22 (26.8)	38 (46.3)	22 (26.8)

* One professional was excluded from the analysis due to incomplete responses on the ProQOL-BR;

** ProQOL-BR = Professional Quality of Life Scale - BR.

The association analysis between occupational stress and compassion fatigue (Table 3) suggests that professionals with occupational stress are 166.7% more likely to develop compassion fatigue (OR = 2.668; 95%CI: 0.799-8.898). Although these results are not conclusive (p > 0.05), there is evidence that occupational stress may be related to compassion fatigue.

**Table 3:** Association analysis between occupational stress and compassion fatigue, Rio Grande, Rio Grande do Sul, Brazil, (N = 82)^*^

Variables	Occupational Stress		Odds Ratio (95% CI)	*p* value^**^
	Yes n(%)	No n(%)		
Compassion Fatigue			2.668 (0.799 - 8.898)	0.096
Yes	7 (53.8)	6 (46.2)		
No	21 (30.4)	48 (69.6)		

* One professional was excluded from the analysis due to incomplete responses on the ProQOL-BR;

** Fisher’s exact test.

Regarding work engagement, Cronbach’s alpha coefficients indicated good reliability: absorption (α = 0.87), vigor (α = 0.88), and dedication (α = 0.70). Nurses demonstrated high levels of dedication (4.6 ± 1.0), absorption (4.2 ± 1.0), and vigor (4.4 ± 1.1) (data not shown in table).

The comparative analysis of the professional quality of life subscales and the dimensions of work engagement between professionals with and without occupational stress (Table 4) revealed some significant differences. The results showed that professionals with occupational stress had significantly higher levels of burnout (mean = 53.8; p = 0.013) and STS (mean = 53.0; p = 0.047) compared to professionals without occupational stress. Conversely, no significant differences were found between the groups in terms of CS (p = 0.888) or in the dimensions of work engagement (p > 0.05). However, there was a slight decrease in levels of dedication, absorption, and vigor among professionals with occupational stress compared to those without occupational stress.

**Table 4:** Comparative analysis of the scores on the professional quality of life subscales and the dimensions of work engagement between professionals with and without occupational stress, Rio Grande, Rio Grande do Sul, Brazil, 2023 (N = 83)

Variables	Mean Score (Standard Deviation)	95% CI	t-test
Compassion Satisfaction			
Without Occupational Stress	49.9 (10.6) ^b^	(47.0 - 52.8)	0.888
With Occupational Stress	50.2 (8.8) ^b^	(46.8 - 53.6)	
Burnout			
Without Occupational Stress	48.1 (9.7) ^b^	(45.4 - 50.7)	**0.013**
With Occupational Stress	53.8 (9.6) ^b^	(50.0 - 57.5)	
Secondary Traumatic Stress			
Without Occupational Stress	48.4 (9.2) ^b^	(45.9 - 50.9)	**0.047**
With Occupational Stress	53.0 (10.9) ^b^	(48.8 - 57.3)	
Dedication			
Without Occupational Stress	4.8 (0.9) ^b^	(4.5 - 5.0)	0.158
With Occupational Stress	4.4 (1.3) ^b^	(3.9 - 4.9)	
Absorption			
Without Occupational Stress	4.3 (0.9) ^b^	(4.1 - 4.6)	0.077
With Occupational Stress	3.9 (1.2) ^a^	(3.4 - 4.3)	
Vigor			
Without Occupational Stress	4.5 (1.0) ^b^	(4.2 - 4.7)	0.684
With Occupational Stress	4.3 (1.3) ^b^	(3.8 - 4.9)	

a Low level;

b Medium level.

Nurses who presented compassion fatigue had significantly lower levels of dedication (p = 0.011) and vigor (p = 0.001), indicating a probable loss of work engagement. It is noteworthy, however, that both groups (with and without compassion fatigue) demonstrated high levels of engagement (data not shown in table).

The analyses revealed significant correlations between the dimensions of work engagement (dedication, absorption, and vigor) and CS, while the relationships with STS and BO followed an inverse pattern (Table 5).

**Table 5:** Correlations between occupational stress, dimensions of work engagement, and ProQOL-BR dimensions, Rio Grande, Rio Grande do Sul, Brazil, 2023 (N = 82)^**^

	Compassion Satisfaction	Secondary Traumatic Stress	Burnout	Occupational Stress	Absorption	Vigor
Dedication	0.504^***^ (<0.001)	-0.374^**^ (0.001)	-0.563^***^ (<0.001)	-0.366^***^ (0.001)	0.498^***^ (<0.001)	0.781^***^ (<0.001)
Absorption	0.240^**^ (0.035)	-0.051 (0.660)	-0.305^***^ (0.007)	-0.323^***^ (0.004)		0.507^***^ (<0.001)
Vigor	0.479^***^ (<0.001)	-0.319^***^ (0.004)	-0.581^***^ (<0.001)	-0.265^**^ (0.018)		

Pearson’s correlation test;

* One professional was excluded from the analysis due to incomplete responses on the ProQOL-BR;

** Correlation significant at the 0.05 level;

*** Correlation significant at the 0.01 level.

CS showed a positive correlation with dedication (r = 0.540; p < 0.001) and vigor (r = 0.479; p < 0.001), indicating that professionals who experience pleasure and fulfillment from helping others tend to have more energy and motivation. In contrast, STS showed a negative correlation with dedication (r = -0.374; p = 0.001) and vigor (r = -0.319; p = 0.004), demonstrating that exposure to others’ suffering reduces emotional commitment and the energy invested in work.

BO showed stronger negative correlations with dedication (r = -0.563; p < 0.001) and vigor (r = -0.581; p < 0.001), indicating that exhaustion directly impairs engagement. Occupational stress showed weaker but still significant negative correlations with dedication (r = -0.366; p = 0.001) and vigor (r = -0.265; p = 0.018), suggesting that adverse working conditions affect engagement, though to a lesser extent than BO and traumatic stress.

Additionally, the strong positive correlation between dedication and vigor (r = 0.781; p < 0.001) reinforces the idea that engagement strengthens professionals’ capacity to cope with occupational challenges.

## DISCUSSION

The study identified a positive correlation between occupational stress and compassion fatigue among nurses in a university hospital, with prevalences of 34.9% and 15.9%, respectively. Professionals with occupational stress were more likely to develop compassion fatigue, although the results were not statistically conclusive. Nevertheless, the nurses demonstrated high levels of engagement, with elevated means in dedication, absorption, and vigor, indicating that even when engaged, they are susceptible to emotional strain, especially under occupational stress.

Sociodemographic factors, work structure, and professional experience directly influence the development of conditions such as secondary traumatic stress, BO, and compassion fatigue^(3,4,7,16)^. A 2019 study in Saudi Arabia confirmed the relationship between demographic characteristics and compassion fatigue among nurses in public hospitals^(16)^, corroborating our findings regarding high levels of BO and secondary traumatic stress.

Although nurses reported high CS - reflecting fulfillment and success in their work - they experienced significant psychological and emotional strain, which can compromise their health and work capacity^(5)^. The intense emotional burden of caring for critically ill or terminal patients contributes to exhaustion and depersonalization, which are characteristic of BO. Additionally, compassion fatigue may coexist with BO, as both share common stressors in the healthcare work environment^(17)^.

Assessing CS is crucial, as it reflects the degree of fulfillment derived from work. Emotional exhaustion caused by STS can impair the ability to provide optimal care^(2,3)^. With the COVID-19 pandemic, nurses’ work environments became even more complex and risky. The health crisis reduced the number of healthcare professionals due to deaths, leave related to comorbidities, and attrition, thereby exacerbating the shortage of nurses^(18,19)^.

This scenario intensified stressors, destabilizing the mental health and well-being of professionals, and increasing the risk of occupational stress, BO, secondary traumatic stress, and compassion fatigue^(2)^. Fear of infection, social isolation, and the loss of colleagues and family members further deteriorated mental health and undermined work engagement^(20)^.

The pandemic underscored the importance of appropriate emotional and institutional support, such as psychological support programs and strategies to foster resilience. These interventions are essential to mitigate the negative impacts on work engagement, strengthen mental well-being, and increase CS^(21)^.

More than one-third of the nurses evaluated showed significant levels of occupational stress, associated with high emotional demands and poor working conditions. These factors contribute to psychological distress and exhaustion, with 24.4% of professionals experiencing occupational stress and compassion fatigue simultaneously. During the pandemic, precarious working conditions amplified these impacts, with consequences that may persist for years^(22,23)^.

The results are consistent with studies conducted in Ireland and China, which confirm that work overload and unfavorable conditions promote occupational stress and facilitate the development of BO and secondary traumatic stress^(23)^.

The nurses demonstrated high levels in all dimensions of work engagement, indicating energy, willingness, and connection to their professional activities, which help them cope with the challenges of clinical practice^(9)^. High engagement is directly associated with job performance, reinforcing nurses’ roles in patient care and hospital unit management. These results suggest that despite exhausting factors, the hospital’s structure and organization provide conditions that support the delivery of comprehensive patient care^(2,24)^.

A slight tendency toward reduced engagement was observed as a result of occupational stress, possibly related to stressors, BO, and secondary traumatic stress, which may lead to compassion fatigue and affect the quality of care^(2,25)^. Occupational stress undermines engagement by reducing professionals’ energy and willingness to work^(6,24)^, while CS functions as a protective factor, fostering resilience and greater involvement, which positively impacts nurses’ well-being and mental health^(26)^.

The emotional overload associated with STS interferes with engagement, increasing the risk of psychological exhaustion and reducing energy and dedication^(27)^. Therefore, institutional strategies that promote CS and reduce stressors, BO, and STS are essential for preserving engagement and professional well-being^(28)^. These findings reinforce the Job Demands-Resources (JD-R) model, which emphasizes the importance of emotional, social, and organizational resources in reducing the impact of stressors and increasing engagement^(29)^.

Encouraging engagement through appreciation, recognition, and emotional support can increase professional commitment, improve care quality, and enhance problem-solving efficiency. However, this requires the implementation of measures such as adequate team staffing and psychological support to help professionals cope with human suffering and end-of-life care^(18,30)^.

### Study limitations

This study has important limitations. The use of a convenience sample compromises the generalizability of the results, as participants were not randomly selected, introducing selection bias and limiting applicability to other contexts.

With a response rate of 58.9%, some losses may have excluded a representative portion of the target population. Factors such as the hospital’s organizational profile, specific characteristics of the nurses, and working conditions may have influenced the results. Moreover, the analysis did not control for potential confounding factors such as age, years of professional experience, and work shifts, which could have distorted some associations.

As a cross-sectional study, it was not possible to establish causality, limiting the interpretation of correlations. These points reinforce the need for future research with probabilistic sampling across multiple hospital settings.

### Contributions to Nursing, Health, or Public Policy

This study provides relevant information for the development of institutional strategies to promote nurses’ well-being. It presents a pioneering analysis of the relationship between occupational stress, compassion fatigue, and work engagement in a university hospital, with data that may inform occupational health policies. The use of validated instruments ensured the accuracy of variable measurement, highlighting the resilience of professionals even in challenging contexts. Additionally, the study contributes to the understanding of compassion fatigue among Brazilian nurses, supporting interventions aimed at mitigating emotional strain in hospital settings.

## CONCLUSIONS

The study identified a high prevalence of occupational stress and compassion fatigue among nurses at a university hospital, even before the COVID-19 pandemic. Despite good levels of engagement, professional quality of life was significantly affected. These findings underscore the need for strategic action by hospital administrators, healthcare professionals, and organizational leaders to prevent and mitigate the effects of occupational stress and compassion fatigue.

With the worsening of conditions during the pandemic, the need for strategic responses became even more evident. Public policies and occupational health programs, such as appropriate staff-to-patient ratios, psychological support, and professional recognition, are essential to reducing the impact of stress and promoting well-being.

The results contribute to the growing body of knowledge on the relationship between occupational stress, compassion fatigue, and work engagement, providing a foundation for future investigations in the field of occupational health.

## Data Availability

The research data are available only upon request.
