# Supplementary material for: Interactions between occupational stress, compassion fatigue, and work engagement among nurses
Source: Rev Bras Enferm. 2025 Nov 7;78(4):e20240555. doi: 10.1590/0034-7167-2024-0555 (PMC12594483; doi:10.1590/0034-7167-2024-0555)
Supplement: Translation

# Translation: Interações entre estresse ocupacional, fadiga por compaixão e engajamento no trabalho em enfermeiros

**DOI:** 10.1590/0034-7167-2024-0555pt

**Authors:** Luciano Garcia Lourenção, Francielle Garcia da Silva, José Gustavo Monteiro Penha, Daniela Menezes Galvão, Vagner Ferreira do Nascimento, Natália Sperli Geraldes Marin dos Santos Sasaki, Francisco Rosemiro Guimarães Ximenes, Luiz Antônio Alves de Menezes

**Affiliations:** I Universidade Federal do Rio Grande. Rio Grande, Rio Grande do Sul, Brasil; II Universidade do Estado do Mato Grosso. Barra do Bugres, Mato Grosso, Brasil; III Faculdade de Medicina de São José do Rio Preto. São José do Rio Preto, São Paulo, Brasil; IV Universidade Estadual Vale do Acaraú. Sobral, Ceará, Brasil; V Universidade Federal de Ouro Preto. Ouro Preto, Minas Gerais, Brasil

**Keywords:** Estresse Ocupacional, Fadiga de Compaixão, Engajamento no Trabalho, Enfermeiras e Enfermeiros, Hospitais.

## Abstract

**Objetivos:**

analisar os níveis de fadiga por compaixão, estresse ocupacional e engajamento no trabalho e sua correlação, em enfermeiros de um hospital universitário.

**Métodos:**

estudo transversal com 83 enfermeiros, realizado em 2019, utilizando escalas validadas. Aplicaram-se testes descritivos, de confiabilidade e de correlação (p≤0,05).

**Resultados:**

prevalência de 34,9% de estresse ocupacional e 15,9% de fadiga por compaixão; altos níveis de dedicação (4,6±1,0), absorção (4,2±1,0) e vigor (4,4±1,1). Enfermeiros com estresse ocupacional tinham maior probabilidade de fadiga por compaixão (OR=2,668; IC95%: 0,799-8,898), níveis elevados de burnout (média=53,8; p=0,013) e estresse traumático secundário (média=53,0; p=0,047). Profissionais com fadiga apresentaram redução na dedicação (p=0,011) e no vigor (p=0,001).

**Conclusões:**

a relação entre estresse ocupacional e fadiga por compaixão aumenta o risco de burnout, estresse traumático secundário e reduz a dedicação e o vigor, destacando a necessidade de medidas institucionais para promover o bem-estar, especialmente no contexto pós-pandêmico, marcado por sobrecarga, escassez de recursos e sofrimento.

## INTRODUÇÃO

O ambiente hospitalar apresenta desafios físicos e psicossociais para os enfermeiros, que lidam com sofrimento dos pacientes, altas cargas de trabalho e pressão por decisões rápidas e precisas. Essas situações podem desencadear fadiga por compaixão e estresse, comprometendo o engajamento e afetando o bem-estar dos profissionais e a qualidade dos serviços^(1,2)^.

A fadiga por compaixão resulta da exaustão física, emocional e mental associada ao cuidado de pessoas em sofrimento intenso, causando desregulação biológica, psicológica e social^(3)^. Conforme o modelo de Qualidade de Vida Profissional, ela ocorre quando altos níveis de burnout e estresse traumático secundário se combinam com baixa satisfação por compaixão. Enquanto o burnout reflete esgotamento e ineficiência, o estresse traumático secundário decorre da exposição contínua ao sofrimento, gerando distúrbios do sono e memórias perturbadoras. Por outro lado, a satisfação por compaixão traduz o prazer em ajudar de forma significativa^(2,4)^.

No Brasil, a fadiga por compaixão é observada entre enfermeiros da Atenção Primária^(5)^ e profissionais de unidades críticas, com maior incidência entre técnicas de enfermagem, mulheres e jovens^(4)^.

O estresse ocupacional refere-se à resposta física, emocional e comportamental a demandas excessivas no trabalho^(6)^. Quando prolongado, pode resultar em ansiedade, depressão, distúrbios psicossomáticos, além de afetar a qualidade de vida e o desempenho profissional^(7,8)^. Essa condição biopsicossocial é complexa e gera grande preocupação com a saúde dos profissionais, especialmente devido à sua alta prevalência entre enfermeiros^(6,7)^.

O engajamento no trabalho é essencial para entender a motivação e o desempenho dos profissionais, representando um estado mental positivo caracterizado por vigor, dedicação e absorção nas atividades. Esse conceito está diretamente ligado a resultados positivos, tanto para os trabalhadores quanto para as organizações. O vigor reflete a energia e disposição que os profissionais sentem no desempenho de suas funções, enquanto a dedicação envolve um profundo senso de propósito e motivação. A absorção, por sua vez, refere-se à imersão completa nas tarefas, em que o profissional perde a noção do tempo, demonstrando alta concentração. Essas três dimensões interagem entre si e são indicadores essenciais do bem-estar dos funcionários, impactando a qualidade dos serviços e a eficácia organizacional^(6,9)^.

O desenvolvimento da fadiga por compaixão e do estresse ocupacional pode impactar o engajamento no trabalho dos enfermeiros^(2,7)^, tornando fundamental a avaliação dessas condições. Estudos são essenciais para compreender os desafios enfrentados por esses profissionais e para elaborar estratégias que auxiliem na prevenção e resolução dessas questões. Embora essas condições sejam comuns no setor de saúde global, fatores como localização, setor hospitalar de atuação e baixos investimentos em saúde influenciam significativamente o aumento ou a redução desses problemas. Ademais, dificuldades já presentes antes da pandemia de COVID-19, como a precariedade das condições de trabalho, jornadas excessivas, exaustão e sofrimento, foram agravadas no contexto pandêmico. O medo de lidar com uma doença desconhecida também impactou diretamente a saúde mental e física dos enfermeiros^(2,10)^.

Além disso, questões como baixa remuneração e falta de reconhecimento profissional, que há tempos afetam esses trabalhadores, podem ter se intensificado durante a pandemia^(11)^. Portanto, é relevante desenvolver estudos locais e em diferentes instituições hospitalares, considerando as especificidades de cada contexto. Essas pesquisas podem subsidiar o desenvolvimento de medidas de enfrentamento direcionadas, mais adequadas e eficazes para melhorar as condições de trabalho e o bem-estar dos enfermeiros.

Com base no exposto, este estudo teve como questão norteadora: Qual a correlação entre estresse ocupacional, fadiga por compaixão e engajamento no trabalho em enfermeiros que trabalham em ambiente hospitalar? A hipótese adotada é que os profissionais de enfermagem que atuam em unidades hospitalares enfrentam estresse ocupacional e fadiga por compaixão, que podem reduzir os níveis de engajamento no trabalho.

## OBJETIVOS

Analisar os níveis de fadiga por compaixão, estresse ocupacional e engajamento no trabalho e sua correlação, em enfermeiros de um hospital universitário.

## MÉTODOS

### Aspectos éticos

Atendendo aos preceitos ético-legais relacionados à pesquisa com seres humanos, o estudo foi aprovado pelo Comitê de Ética em Pesquisa da Universidade Federal do Rio Grande.

### Desenho, período e local do estudo

Estudo transversal, descritivo e correlacional, realizado entre setembro e dezembro de 2019, com enfermeiros que trabalhavam em um hospital universitário de médio porte, vinculado à Empresa Brasileira de Serviços Hospitalares (EBSERH) e localizado no extremo sul do Brasil. A ferramenta *Strengthening the Reporting of Observational Studies in Epidemiology* (STROBE) foi utilizada para nortear a pesquisa.

### População e amostra: critérios de inclusão e exclusão

A população do estudo foi composta por todos os enfermeiros que trabalhavam no hospital universitário há, pelo menos, seis meses. Esse período foi estabelecido pelos pesquisadores, considerando que, em seis meses, os profissionais já se encontram familiarizados com o ambiente de trabalho. Foram excluídos os profissionais em licença médica ou de férias no período da coleta, resultando em uma população estimada de 141 enfermeiros.

A amostra foi selecionada por conveniência, um método justificado pela acessibilidade e disponibilidade dos participantes, que permitiu a coleta de dados de forma prática e viável dentro do período delimitado para a pesquisa. Essa metodologia possibilitou a inclusão de uma proporção significativa dos profissionais da instituição, garantindo representatividade da equipe de enfermagem do hospital universitário analisado. Todos os profissionais foram convidados, e aqueles que devolveram os instrumentos preenchidos foram incluídos na amostra final, composta por 83 enfermeiros (58,9% da população). Não houve recusa formal em participar do estudo.

### Protocolo do estudo

Foram utilizados quatro instrumentos autoaplicáveis: um questionário sobre características sociodemográficas e profissionais (como formação acadêmica, sexo, idade, renda, estado civil, vínculo empregatício, turno de trabalho, tempo de atuação, horas de sono, vínculos laborais adicionais e prática de atividade física); a Escala de Estresse no Trabalho (EET)^(12)^; a versão brasileira da *Professional Quality of Life Scale* (ProQOL-BR)^(13)^; e a versão resumida da *Utrecht Work Engagement Scale* (UWES-9)^(14)^.

A EET, composta por 23 itens, avalia situações de estresse no trabalho. Sua validação no Brasil apresentou alta consistência interna (α = 0,91; Kaiser-Meyer-Olkin - KMO = 0,91)^(6,12)^. A ProQOL-BR mede Satisfação por Compaixão (SC), Burnout (BO) e Estresse Traumático Secundário (ETS), com 30 questões distribuídas em três categorias (10 itens cada). Sua versão brasileira demonstrou boa fatorabilidade (KMO = 0,861) e consistência interna (SC = 0,81; BO = 0,83; ETS = 0,76)^(4,13)^.

A UWES-9 avalia engajamento no trabalho por meio das dimensões Vigor, Dedicação e Absorção, apresentando robustas propriedades psicométricas no Brasil (α = 0,93; CFI- Comparative Fit Index = 0,97; TLI - Tucker-Lewis Index = 0,98)^(9,14)^.

Os dados foram coletados, presencialmente, por um enfermeiro pós-graduando, devidamente capacitado. Os profissionais foram abordados em seus setores de trabalho, individualmente ou em grupos. Houve a apresentação do objetivo da pesquisa e o convite aos profissionais para participarem do estudo; a entrega do Termo de Consentimento Livre e Esclarecido (TCLE) e dos instrumentos de coleta. Foi estabelecido um prazo para que respondessem aos instrumentos até o próximo plantão. Após o término desse prazo, o pós-graduando voltava aos setores para recolher os instrumentos preenchidos, que eram entregues sem identificação. Caso os profissionais esquecessem ou alegassem não ter tido tempo para responder aos instrumentos, o prazo era estendido até o próximo plantão.

Para minimizar as perdas amostrais e evitar viés de seleção, foram adotadas estratégias como o convite pessoal aos enfermeiros durante seus turnos de trabalho e a definição de prazos flexíveis, permitindo que os profissionais respondessem aos questionários até o próximo plantão, com extensões adicionais, caso necessário. Essas medidas buscaram aumentar a adesão e reduzir o impacto das perdas amostrais.

### Análise dos resultados e estatística

Os dados foram inseridos duas vezes com o objetivo de detectar inconsistências ou erros. Para avaliar se os dados seguiam uma distribuição normal, utilizou-se o teste de Kolmogorov-Smirnov, com os seguintes resultados: Estresse Ocupacional (p = 0,470), Dedicação (p = 0,230), Absorção (p = 0,326), Vigor (p = 0,400), Satisfação por Compaixão (p = 0,061), Burnout (p = 0,788) e Estresse Traumático Secundário (p = 0,374). A análise da confiabilidade das medidas dos construtos indicou que os valores do Coeficiente Alfa de Cronbach variaram de 0,70 a 0,92, o que evidencia a confiabilidade dos resultados^(9)^. As variáveis sociodemográficas e profissionais foram utilizadas para descrever o perfil dos enfermeiros.

Para avaliar o estresse ocupacional, a pontuação da escala foi calculada pela média dos 23 itens. Esse escore varia de 1 a 5 e, conforme preconizado pelo instrumento, quanto maior o valor obtido, maior o nível de estresse. Valores médios iguais ou superiores a 2,5 indicam um nível de estresse significativo^(6)^. Posteriormente, a variável foi classificada de forma dicotômica em “com estresse” (EET ≥ 2,5) ou “sem estresse” (EET < 2,5).

A análise da fadiga por compaixão foi realizada por meio da determinação dos escores gerais das subescalas da ProQOL-BR^(2,4)^. Os escores brutos das dimensões da ProQOL-BR (SC: Satisfação com a Compaixão; BO: Burnout; e ETS: Estresse Traumático Secundário) foram transformados em *z-scores*, por meio da análise descritiva, selecionando o comando “salvar valores padronizados como variáveis”. Em seguida, os *z-scores* foram transformados em *t-scores*, por meio das fórmulas: (ZSC*10)+50; (ZBO*10)+50; (ZETS*10)+50, possibilitando a comparação das três dimensões^(4)^.

A pontuação das subescalas foi computada e, posteriormente, classificada conforme o percentil 75 da distribuição dos valores. Os profissionais que tiveram uma pontuação igual ou superior ao percentil 75 em cada subescala foram considerados com alto nível de burnout ou de estresse traumático secundário. Posteriormente, a fadiga por compaixão foi definida como a junção do alto nível de burnout e alto nível de estresse traumático secundário^(4)^.

Para a avaliação do engajamento no trabalho, foram calculados os escores das dimensões da escala UWES, seguindo o modelo estatístico proposto no *Manual Preliminar UWES*. Foi apresentada a média e o desvio padrão para cada dimensão da escala. Após o cálculo dos escores, os valores obtidos foram classificados de acordo com a decodificação do manual, em que valores de 0 a 0,99 indicam um engajamento muito baixo, valores de 1 a 1,99 indicam um engajamento baixo, valores de 2 a 3,99 indicam um engajamento médio, valores de 4 a 4,99 indicam um engajamento alto, e valores de 5 a 6 indicam um engajamento muito alto^(9)^.

A análise de associação entre estresse ocupacional e fadiga por compaixão foi realizada por meio do cálculo do *odds ratio* (OR), utilizando o teste exato de Fisher. As comparações entre os escores médios das diferentes dimensões das escalas foram realizadas pelos testes *t*-Student. O nível de significância adotado foi de 5% (p≤0,05).

O teste de correlação de Pearson (r) foi aplicado para analisar a correlação entre os valores das diferentes escalas, considerando-se correlação fraca para valores de r até 0,39, moderada para valores entre 0,40 e 0,69, e forte para valores iguais ou superiores a 0,70^(9)^.

## RESULTADOS

Entre os 83 profissionais avaliados, houve predomínio do sexo feminino (75,9%), faixa etária de 26 a 35 anos (50,6%), casados ou em união estável (57,8%), servidores públicos estatutários (97,6%) e renda familiar de dois a cinco salários mínimos (57,1%). A maioria dos profissionais exercia atividades administrativas e assistenciais (49,4%), trabalhava predominantemente no período noturno (38,6%), praticava atividade física (62,7%), referiu dormir entre seis e oito horas diárias (68,7%) e tinha entre dois e 10 anos (39,8%) ou entre 10 e 20 anos (32,1%) de experiência profissional (Tabela 1).

**Tabela 1:** Características sociodemográficas e profissionais dos enfermeiros, Rio Grande, Rio Grande do Sul, Brasil, 2023 (N = 83)

Variáveis	n	%
Sexo		
Masculino	20	24,1
Feminino	63	75,9
Faixa Etária (anos)^*^		
26 a 35 (formação profissional)	42	50,6
36 a 50 (maturidade profissional)	29	34,9
51 a 60 (desaceleração profissional)	6	7,2
61 ou mais (aposentadoria)	1	1,2
Não respondeu	5	6,0
Estado Civil		
Casado/União Estável	48	57,8
Solteiro	22	26,5
Separado	8	9,6
Não respondeu	5	6,0
Tipo de Vínculo Empregatício		
Servidores Públicos Estatutários	81	97,6
Empregados regidos pela Consolidação das Leis do Trablaho	2	2,4
Renda Familiar (salários mínimos^**^)		
De dois a cinco	7	8,4
De seis a 10	56	67,5
Mais de 10	20	24,1
Tipo de Atividade		
Assistencial	30	36,4
Administrativa	12	14,5
Administrativa e Assistencial	41	49,4
Turno de Trabalho		
Manhã	19	22,9
Tarde	19	22,9
Noturno	32	38,6
Integral Diurno	13	15,7
Pratica Atividade Física		
Sim	52	62,7
Não	31	37,3
Horas Diárias de Sono		
Menos de 6	23	27,7
De 6 a 8	57	68,7
Mais de 8	3	3,6
Tempo de Experiência Profissional		
≤ 2 anos	6	7,2
> 2 e ≤ 10 anos	33	39,8
> 10 e ≤ 20 anos	26	32,1
> 20 anos	14	16,9
Não respondeu	4	4,8

* Classificação das faixas etárias, segundo Machado et al., 2016^(15)^;

** Valor do salário mínimo: R$ 998,00 / USD 236,29 (1 USD = R$4,2235).

O coeficiente Alfa de Cronbach evidenciou que a escala de estresse no trabalho apresentou alta confiabilidade (α = 0,92). Os resultados mostraram que 29 enfermeiros (34,9%) apresentaram escores indicativos de estresse significativo (≥ 2,5).

Na avaliação da qualidade de vida profissional (Tabela 2), observou-se que 24,4% dos enfermeiros apresentaram nível alto de satisfação por compaixão, 25,6% tinham nível alto de burnout e 26,8% nível alto de estresse traumático secundário. Em contrapartida, 24,4% apresentaram nível baixo de satisfação por compaixão, 50,0% nível médio de burnout e 46,3% nível médio de estresse traumático secundário. Além disso, 13 enfermeiros (15,9%) apresentaram fadiga por compaixão (alto nível de burnout associado a um alto nível de estresse traumático secundário).

**Tabela 2:** Pontos de corte da ProQol-BR e frequências de enfermeiros por nível de classificação das dimensões da qualidade de vida profissional, Rio Grande, Rio Grande do Sul, Brasil (N = 82)^*^

Dimensões da ProQol-BR^**^	Alfa de Cronbach	Pontos de Corte - Percentis (t scores)			Níveis de Classificação n (%)		
		25	50	75	Baixo	Médio	Alto
Satisfação por compaixão	0,84	42,9	50,5	58,0	20 (24,4)	42 (51,2)	20 (24,4)
*Burnout*	0,71	42,8	48,8	58,0	20 (24,4)	41 (50,0)	21 (25,6)
Estresse traumático secundário	0,77	42,8	50,0	58,6	22 (26,8)	38 (46,3)	22 (26,8)

* Um profissional foi excluído da análise por incompletude das respostas da ProQol-BR;

** ProQol-BR = Professional Quality of Life Scale - BR.

A análise de associação entre estresse ocupacional e fadiga por compaixão (Tabela 3) sugere que os profissionais com estresse ocupacional têm uma chance 166,7% maior de desenvolver fadiga por compaixão (OR=2,668; IC95%: 0,799-8,898). Embora esses resultados não sejam conclusivos (p>0,05), há evidências de que o estresse ocupacional pode estar relacionado à fadiga por compaixão.

**Tabela 3:** Análise de associação entre estresse ocupacional e fadiga por compaixão, Rio Grande, Rio Grande do Sul, Brasil (N = 82)^*^

Variáveis	Estresse Ocupacional		Odds Ratio (IC 95%)	Valor de *p* ^**^
	Sim n(%)	Não n(%)		
Fadiga por Compaixão			2,668 (0,799 - 8,898)	0,096
Sim	7 (53,8)	6 (46,2)		
Não	21 (30,4)	48 (69,6)		

* Um profissional foi excluído da análise por incompletude das respostas da ProQol-BR;

** Teste exato de Fisher.

Em relação ao engajamento no trabalho, os coeficientes de Alfa de Cronbach indicaram boa confiabilidade: absorção (α = 0,87), vigor (α = 0,88) e dedicação (α = 0,70). Os enfermeiros apresentaram altos níveis de dedicação (4,6 ± 1,0), absorção (4,2 ± 1,0) e vigor (4,4 ± 1,1) (dados não apresentados em tabela).

A análise comparativa das subescalas da qualidade de vida profissional e das dimensões do engajamento no trabalho entre os profissionais com e sem estresse ocupacional (Tabela 4) evidenciou algumas diferenças significativas. Os resultados mostraram que profissionais com estresse ocupacional apresentaram níveis significativamente mais altos de burnout (média = 53,8; p = 0,013) e de estresse traumático secundário (média = 53,0; p = 0,047), em comparação aos profissionais sem estresse ocupacional. Em contrapartida, não foram encontradas diferenças significativas entre os grupos nos níveis de satisfação por compaixão (p = 0,888) e nas dimensões do engajamento no trabalho (p > 0,05). Houve, contudo, uma leve diminuição dos níveis de dedicação, absorção e vigor entre os profissionais com estresse ocupacional, em comparação àqueles sem estresse ocupacional.

**Tabela 4:** Análise comparativa dos escores das subescalas da qualidade de vida profissional e das dimensões do engajamento no trabalho entre profissionais com e sem estresse ocupacional, Rio Grande, Rio Grande do Sul, Brasil, 2023 (N = 83)

Variáveis	Escore Médio (desvio padrão)	IC95%	Test t
Satisfação por Compaixão			
Sem Estresse Ocupacional	49,9 (10,6) ^b^	(47,0 - 52,8)	0,888
Com Estresse Ocupacional	50,2 (8,8) ^b^	(46,8 - 53,6)	
Burnout			
Sem Estresse Ocupacional	48,1 (9,7) ^b^	(45,4 - 50,7)	**0,013**
Com Estresse Ocupacional	53,8 (9,6) ^b^	(50,0 - 57,5)	
Estresse Traumático Secundário			
Sem Estresse Ocupacional	48,4 (9,2) ^b^	(45,9 - 50,9)	**0,047**
Com Estresse Ocupacional	53,0 (10,9) ^b^	(48,8 - 57,3)	
Dedicação			
Sem Estresse Ocupacional	4,8 (0,9) ^b^	(4,5 - 5,0)	0,158
Com Estresse Ocupacional	4,4 (1,3) ^b^	(3,9 - 4,9)	
Absorção			
Sem Estresse Ocupacional	4,3 (0,9) ^b^	(4,1 - 4,6)	0,077
Com Estresse Ocupacional	3,9 (1,2) ^a^	(3,4 - 4,3)	
Vigor			
Sem Estresse Ocupacional	4,5 (1,0) ^b^	(4,2 - 4,7)	0,684
Com Estresse Ocupacional	4,3 (1,3) ^b^	(3,8 - 4,9)	

a Nível baixo;

b Nível médio.

Os enfermeiros que apresentavam fadiga por compaixão tinham níveis significativamente menores de dedicação (p = 0,011) e vigor (p = 0,001), evidenciando uma provável perda do engajamento no trabalho. Destaca-se, no entanto, que ambos os grupos (com e sem fadiga por compaixão) apresentaram níveis elevados de engajamento (dados não apresentados em tabela).

As análises revelaram correlações significativas entre as dimensões do engajamento no trabalho (dedicação, absorção e vigor) e a satisfação por compaixão, enquanto as relações com o estresse traumático secundário e o burnout apresentaram padrão inverso (Tabela 5).

**Tabela 5:** Correlações entre o estresse ocupacional, as dimensões do engajamento no trabalho e as dimensões da ProQOL-BR, Rio Grande, Rio Grande do Sul, Brasil, 2023 (N = 82)^*^

	Satisfação por Compaixão	Estresse Traumático Secundário	Burnout	Estresse Ocupacional	Absorção	Vigor
Dedicação	0,504^***^ (<0,001)	-0,374^**^ (0,001)	-0,563^***^ (<0,001)	-0,366^***^ (0,001)	0,498^***^ (<0,001)	0,781^***^ (<0,001)
Absorção	0,240^**^ (0,035)	-0,051 (0,660)	-0,305^***^ (0,007)	-0,323^***^ (0,004)		0,507^***^ (<0,001)
Vigor	0,479^***^ (<0,001)	-0,319^***^ (0,004)	-0,581^***^ (<0,001)	-0,265^**^ (0,018)		

Teste de correlação de Pearson;

* Um profissional foi excluído da análise por incompletude das respostas da ProQol-BR;

** Correlação significante no nível 0,05;

*** Correlação significante no nível 0,01.

A satisfação por compaixão mostrou correlação positiva com dedicação (r = 0,540; p < 0,001) e vigor (r = 0,479; p < 0,001), indicando que profissionais que sentem prazer e realização ao ajudar tendem a ter mais energia e motivação. Em contrapartida, o estresse traumático secundário teve correlação negativa com dedicação (r = -0,374; p = 0,001) e vigor (r = -0,319; p = 0,004), evidenciando que a exposição ao sofrimento alheio reduz o comprometimento emocional e a energia investida no trabalho.

O burnout apresentou correlações negativas mais fortes com dedicação (r = -0,563; p < 0,001) e vigor (r = -0,581; p < 0,001), mostrando que o esgotamento prejudica diretamente o engajamento. O estresse ocupacional teve correlações negativas mais fracas, mas significativas, com dedicação (r = -0,366; p = 0,001) e vigor (r = -0,265; p = 0,018), sugerindo que condições adversas no trabalho afetam o engajamento, mas em menor grau do que o burnout e o estresse traumático.

Além disso, a forte correlação positiva entre dedicação e vigor (r = 0,781; p < 0,001) reforça que o engajamento fortalece a capacidade dos profissionais de lidar com desafios ocupacionais.

## DISCUSSÃO

O estudo identificou uma correlação positiva entre estresse ocupacional e fadiga por compaixão em enfermeiros de um hospital universitário, com prevalências de 34,9% e 15,9%, respectivamente. Profissionais com estresse ocupacional apresentaram maior propensão à fadiga por compaixão, embora sem resultados estatisticamente conclusivos. Apesar disso, os enfermeiros mostraram altos níveis de engajamento, com médias elevadas em dedicação, absorção e vigor, indicando que, mesmo engajados, estão suscetíveis ao desgaste emocional, especialmente sob estresse ocupacional.

Fatores sociodemográficos, estrutura laboral e experiência profissional influenciam diretamente o desenvolvimento de condições como estresse traumático secundário, burnout e fadiga por compaixão^(3,4,7,16)^. Um estudo de 2019 na Arábia Saudita confirmou a relação entre características demográficas e fadiga por compaixão em enfermeiros de hospitais públicos^(16)^, corroborando nossos achados sobre altos níveis de burnout e estresse traumático secundário.

Embora os enfermeiros apresentem alta satisfação por compaixão, refletindo contentamento e sucesso no trabalho, eles sofrem importantes desgastes psíquico-emocionais que podem comprometer a saúde e a capacidade laboral^(5)^. A intensa carga emocional do cuidado a pacientes críticos ou terminais contribui para a exaustão e despersonalização, características do burnout. Além disso, a fadiga por compaixão pode coexistir com o burnout, já que ambos compartilham fatores estressores do ambiente de trabalho na saúde^(17)^.

A avaliação da satisfação por compaixão é crucial, pois mede o grau de realização no trabalho. O esgotamento emocional devido ao estresse traumático secundário pode prejudicar a capacidade de oferecer um atendimento ideal^(2,3)^.

Com a pandemia de COVID-19, as unidades de trabalho dos enfermeiros tornaram-se ainda mais complexas e arriscadas. A crise sanitária reduziu o número de profissionais da saúde devido a óbitos, afastamentos por comorbidades e abandono da profissão, ampliando o déficit de enfermeiros^(18,19)^.

Esse cenário agravou os fatores estressores, desestabilizando a saúde mental e o bem-estar dos profissionais, além de aumentar o risco de estresse ocupacional, burnout, estresse traumático secundário e fadiga por compaixão^(2)^. O medo de contaminação, o isolamento social e a perda de colegas e familiares pioraram a saúde mental e comprometeram o engajamento no trabalho^(20)^.

A pandemia destacou a importância de suporte emocional e institucional adequado, como programas de apoio psicológico e estratégias para promover a resiliência. Essas intervenções são fundamentais para mitigar os impactos negativos no engajamento no trabalho, fortalecer o bem-estar mental e aumentar a satisfação por compaixão^(21)^.

Mais de um terço dos enfermeiros avaliados apresentou níveis significativos de estresse ocupacional, associado a altas demandas emocionais e más condições de trabalho. Esses fatores contribuem para o sofrimento psíquico e o esgotamento, com 24,4% dos profissionais manifestando estresse ocupacional e fadiga por compaixão simultaneamente. Durante a pandemia, as condições laborais precárias ampliaram esses impactos, com consequências que podem perdurar por anos^(22,23)^.

Os resultados corroboram estudos realizados na Irlanda e na China, que confirmam que a sobrecarga e as condições desfavoráveis de trabalho promovem o estresse ocupacional e favorecem o desenvolvimento de burnout e estresse traumático secundário^(23)^.

Os enfermeiros apresentaram níveis elevados em todas as dimensões do engajamento no trabalho, evidenciando energia, disposição e conexão com suas atividades laborais, o que os auxilia a lidar com os desafios da prática profissional^(9)^. O alto engajamento está diretamente associado ao desempenho laboral, fortalecendo a atuação dos profissionais na assistência e gestão das unidades hospitalares. Esses resultados sugerem que, apesar de fatores desgastantes, a estrutura e organização do hospital oferecem condições para atender às demandas assistenciais, assegurando cuidado integral aos pacientes^(2,24)^.

Observou-se uma leve tendência à redução do engajamento em função do estresse ocupacional, possivelmente relacionada a estressores, burnout e estresse traumático secundário, que podem levar à fadiga por compaixão e impactar a qualidade do atendimento^(2,25)^. O estresse ocupacional compromete o engajamento ao reduzir a disposição e a energia dos profissionais^(6,24)^, enquanto a satisfação por compaixão funciona como fator protetor, promovendo resiliência e maior envolvimento, o que impacta positivamente o bem-estar e a saúde mental dos enfermeiros^(26)^.

A sobrecarga emocional associada ao estresse traumático secundário interfere no engajamento, aumentando o risco de desgaste psicológico e reduzindo energia e dedicação^(27)^. Assim, estratégias institucionais que promovam a satisfação por compaixão e reduzam estressores, burnout e estresse traumático secundário são fundamentais para preservar o engajamento e o bem-estar dos profissionais^(28)^. Tais achados reforçam o modelo Demanda-Recursos no Trabalho (JD-R), que destaca a importância de recursos emocionais, sociais e organizacionais na redução dos impactos dos estressores e no aumento do engajamento^(29)^.

Estimular o engajamento, por meio de valorização, reconhecimento e suporte emocional, pode aumentar o comprometimento profissional, melhorar a qualidade do atendimento e a eficiência na resolução de problemas. Contudo, para isso, é necessário implementar medidas como dimensionamento adequado das equipes e suporte psicológico para lidar com o sofrimento humano e o enfrentamento da morte^(18,30)^.

### Limitações do estudo

O estudo apresenta limitações importantes. A utilização de uma amostra por conveniência compromete a generalização dos resultados, pois os participantes não foram selecionados aleatoriamente, gerando viés de seleção e restringindo a extrapolação para outros contextos.

Com uma taxa de resposta de 58,9%, houve perdas que podem ter excluído parte representativa da população-alvo. Fatores como o perfil organizacional do hospital, características específicas dos enfermeiros e condições de trabalho podem ter influenciado os resultados. Além disso, a análise não ajustou potenciais fatores de confusão, como idade, tempo de experiência e turnos de trabalho, o que pode ter distorcido algumas associações.

Por ser um estudo transversal, não foi possível estabelecer causalidade, limitando a interpretação das correlações. Esses pontos reforçam a necessidade de pesquisas futuras com amostragem probabilística e abrangendo múltiplos cenários hospitalares.

### Contribuições para a área da Enfermagem, Saúde ou Política Pública

O estudo fornece informações relevantes para a elaboração de estratégias institucionais que promovam o bem-estar dos enfermeiros. Ele apresenta uma análise pioneira da relação entre estresse ocupacional, fadiga por compaixão e engajamento no trabalho em um hospital universitário, com dados que podem orientar políticas de saúde ocupacional.

O uso de instrumentos validados garantiu rigor na mensuração das variáveis, evidenciando a resiliência dos profissionais, mesmo em cenários desafiadores. Além disso, o estudo contribui para o campo da fadiga por compaixão em enfermeiros brasileiros, embasando intervenções que mitiguem o desgaste emocional no ambiente hospitalar.

## CONCLUSÕES

O estudo identificou alta prevalência de estresse ocupacional e fadiga por compaixão entre enfermeiros de um hospital universitário, mesmo antes da pandemia de COVID-19. Apesar dos bons níveis de engajamento, a qualidade de vida profissional foi significativamente impactada. Esses achados destacam a necessidade de ações estratégicas por parte de gestores hospitalares, profissionais de saúde e líderes organizacionais para prevenir e mitigar os efeitos do estresse ocupacional e da fadiga por compaixão.

Com o agravamento das condições durante a pandemia, a necessidade de ações estratégicas tornou-se mais evidente. Políticas públicas e programas de saúde ocupacional, como dimensionamento adequado de equipes, suporte psicológico e reconhecimento profissional, são fundamentais para reduzir o impacto do estresse e promover o bem-estar.

Os resultados contribuem para o aprofundamento do entendimento sobre a relação entre estresse ocupacional, fadiga por compaixão e engajamento no trabalho, fornecendo uma base para futuras investigações no campo da saúde ocupacional.

## Data Availability

Os dados de pesquisa só estão disponíveis mediante solicitação.
